# Towards patient engagement in violence risk assessment and management: a patient perspective

**DOI:** 10.1192/j.eurpsy.2022.710

**Published:** 2022-09-01

**Authors:** T. Lantta, J. Anttila

**Affiliations:** University of Turku, Department Of Nursing Science, UNIVERSITY OF TURKU, Finland

**Keywords:** patient engagement, violence, risk assessment, risk management

## Abstract

**Introduction:**

In current clinical practices, there exist very few methods that allow patients to be truly engaged in violence risk assessment and management. This may hinder an individual’s experience of basic psychological needs; autonomy, competence, and relatedness.

**Objectives:**

To describe patients’ ideas on how they would develop current violence risk assessment and management practices.

**Methods:**

The data collection took place as part of a larger project in one PICU unit specialized in the treatment of patients with psychosis and violent behavior in Finland. Individual interviews were conducted with patients (n=13) and were guided to focus on the development of violence risk assessment and management. The data were analyzed using inductive content analysis.

**Results:**

Patients’ ideas focused on themes related to developing patient engagement and violence risk management methods. Developing patient engagement involved noticing patient’s individuality and collaboration between a patient and staff: for instance, by shared risk assessment and individualized risk management. Developing violence risk management methods included themes about providing alternative risk management methods and developing nursing staff’s work. Suggestions were, for example, related to providing ways how to calm down, having meaningful activities during treatment days, and ensuring the realization of patient’s rights.

**Conclusions:**

Patients having treatment in the PICU unit have clear and concrete ideas on how violence risk assessment and management methods could be developed further. These findings indicate, that patients need to be given a more active role in their care and thus ensure that basic psychological needs are promoted. Funding by Academy of Finland (316206 ) and TYKS foundation.

**Disclosure:**

No significant relationships.

